# Clot Retraction and Its Correlation with the Function of Platelet Integrin α_IIb_β_3_ †

**DOI:** 10.3390/biomedicines11092345

**Published:** 2023-08-23

**Authors:** Daniel Gao, Caroline W. Sun, Angela B. Woodley, Jing-fei Dong

**Affiliations:** 1 Bloodworks Research Institute, 1551 Southlake Ave. E., Seattle, WA 98102, USA; dlga2021@mymail.pomona.edu; 2 Department of Chemistry, Pomona College, Claremont, CA 91711, USA; 3 Section of Cardiovascular Sciences, Department of Medicine, Baylor College of Medicine, Houston, TX 77030, USA; 4 Division of Hematology, Department of Medicine, University of Washington School of Medicine, Seattle, WA 98195, USA

**Keywords:** clot retraction, integrin α_Iib_β_3_, PLA genotypes

## Abstract

Clot retraction results from retractions of platelet filopodia and fibrin fibers and requires the functional platelet α_IIb_β_3_ integrin. This assay is widely used to test the functions of platelets and fibrinogen as well as the efficacy of fibrinolysis. Changes in clot retraction have been found in a variety of hemostatic abnormalities and, more recently, in arterial thrombosis. Despite its broad clinical use and low cost, many aspects of clot retraction are poorly understood. In the present study, we performed two clinical standard clot retraction assays using whole-blood and platelet-rich plasma (PRP) samples to determine how clot retraction correlates with platelet counts and mean volume, the density of α_IIb_β_3_ integrin and PLA genotypes, and plasma fibrinogen levels. We found that clot retraction was affected by platelet counts, but not mean platelet volume. It correlated with the surface density of the integrin α_Iib_β_3_, but not PLA genotypes. These results indicate that clot retraction measures a unique aspect of platelet function and can serve as an additional means to detect functional changes in platelets.

## 1. Introduction

Clot retraction is a process where a blood clot reduces its volume over time. The process is caused by retractions of platelet filopodia, resulting in the retraction of fibrin fibers that attach to these filopodia. The extent of retraction increases in proportion with platelet concentrations [1]. In addition, the functional integrity of platelets is also required because the filopodia retraction depends on an intact platelet cytoskeleton, including actin filaments and microtubules [2,3,4]. Platelets in a clot are likely activated, undergoing cycles of α_IIb_β_3_-dependent protein phosphorylation and dephosphorylation, consistent with the finding that tyrosine phosphatase inhibitors block clot retraction [5,6,7]. As an essential part of the rapid primary hemostatic response, fibrinogen binds activated integrin α_IIb_β_3_ to aggregate platelets in order to develop a hemostatic plug at the site of vascular injury [8]. The platelet integrin α_IIb_β_3_ plays a critical role in clot retraction [9]. Nucleated cells expressing either α_v_β_3_ or α_IIb_β_3_ are also able to retract clots [10]. Interestingly, the antibody D3 to the platelet integrin β_3_ induces fibrinogen binding to α_IIb_β_3_ but completely inhibits clot retraction [11]. In addition, platelets treated with thrombin in the presence of EDTA fail to aggregate but retain their ability to retract [12]. Congruent studies build on this phenomenon, suggesting that the α_IIb_β_3_ binding sites for fibrin during clot contraction differ from their fibrinogen binding sites during platelet aggregation [13]. Experimental evidence also indicates that clot retraction is functionally distinct from platelet granule release [14]. These findings collectively demonstrate that clot retraction measures a unique aspect of platelet function. Equally important is the relationship between clot retraction and fibrinolysis [15]. Whereas clot retraction facilitates internal fibrinolysis, the rate of external fibrinolysis is notably reduced [16]. The latter, especially, can be attributed to the diminished porosity and permeability of contracted clots [17]. Reciprocal studies have suggested that partial fibrinolysis can facilitate clot retraction, potentially by decreasing the rigidity of the fibrin polymers following partial proteolysis [18]. These observations further distinguish clot retraction as possessing a fundamentally distinct set of physiological implications compared to other platelet functions.

Defects in clot retraction have been found in a variety of hemostatic and coagulation abnormalities, including venous thromboembolism, hemophilia A, and stroke [19,20,21]. Clot retraction is severely impaired in the hereditary bleeding disorder Glanzmann’s thrombasthenia caused by defects in the integrin α_IIb_β_3_. Recently, abnormal clot retraction has also been associated with arterial thrombosis. By measuring platelet force development during clot retraction, patients with coronary artery disease presented significantly higher force development and clot elastic modules compared to normal controls [22]. The clinical significance of this finding is that the abnormalities are detectable in patients taking aspirin, which inhibits agonist-induced platelet aggregation [23]. A potential correlation between clot retraction and platelet function is also demonstrated by the recent finding that the PLA2 polymorphism of platelet β_3_ integrin, which increases clot retraction when expressed in CHO cells [24], may result in a high risk of coronary artery disease [25,26,27].

Despite its broad potential as a clinical diagnostic tool, many aspects of clot retraction are poorly understood. Here, we report results that specifically correlated standard clot retraction assays with platelet counts, plasma levels of fibrinogen, the α_IIb_β_3_ density on platelet surfaces, mean platelet volume, and PLA genotypes.

## 2. Material and Methods

### 2.1. Sample Collection

Blood samples from venipuncture were obtained from 53 healthy donors who had been medication-free for at least 7 days and who had no history of thrombotic or bleeding diseases. Platelet aggregation induced by arachidonic acid (Sigma-Aldrich, St. Louis, MO, USA) was performed on each donor to ensure that no aspirin had been taken in the past 72 h. Blood was collected into tubes containing either no anticoagulant, 0.38% sodium citrate, 75 μM of H-Phe-Phe-Arg-chloromethylketone (PPACK, Calbiochem, San Diego, CA, USA), or 100 U/mL of unfractionated heparin (Elkins-Sinn, Inc., Cherry Hill, NJ, USA). Clot retraction was measured within 30 min after blood draw. 

### 2.2. Clot Retraction

Clot retraction was performed in whole-blood and platelet-rich plasma (PRP). Whole-blood clot retraction was induced in the absence of anticoagulants according to a standard clinical method [28]. Briefly, blood (500 μL) was transferred to a 10 × 75 mm disposable glass tube and allowed to stand still for 2 h at 37 °C. The clot was then removed, and the amount of serum was measured to determine the percentage of clot retraction. 

Clot retraction in PRP was measured in the presence of an anticoagulant by a method modified by Owens et al. [29]. Whole blood anticoagulated with citrate, PPACK, or heparin was centrifuged at 150× *g* for 15 min at 27 °C to obtain PRP. Platelet counts were then determined by a Coulter Z2 particle counter (Beckman Coulter, Miami, FL, USA) and diluted to 2–2.5 × 10^8^ platelets/mL using autologous platelet-poor plasma (PPP) obtained by centrifugation of PRP at 900× *g* for 10 min. PRP was mixed with 100 U/mL of human α-thrombin (kindly provided by Dr. JW Fenton of the New York State Department of Health) in a total volume of 600 μL and transferred to a 22 × 72 mm disposable glass tube. After incubation for 2 hrs at 37 °C, the clot was removed, and the amount of serum was measured. 

### 2.3. Quantification of Platelet α_IIb_β_3_ on Platelets

The density of α_IIb_β_3_ integrin on the surface of resting platelets was determined by flow cytometry. We used the phycoerythrin (PE)-conjugated monoclonal antibody P2 (Beckman Coulter, Miami, FL, USA). This antibody recognizes a α_IIb_β_3_ complex, but not its individual subunits on the platelet surface. Five microliters of citrated blood were first incubated for 20 min at room temperature with 70 μL of Ca^2+^- and Mg^2+^-free Tyrode’s buffer (138 mM NaCl, 5.5 mM glucose, 12 mM NaHCO_3_, 0.36 mM Na_2_HPO_4_, 2.9 mM KCl, pH 7.4) containing 1% bovine serum albumin and 5 μg/mL of the P2 antibody. The samples were then fixed with 1 mL of PBS containing 1% paraformaldehyde and analyzed by flow cytometry (Epics XL-MCL, Beckman Coulter). To quantify the numbers of α_IIb_β_3_ molecules on the platelet surface, the mean fluorescence from four sets of PE-conjugated standard beads with the pre-determined amount of PE molecules per bead (Flow Cytometry Standards Corp. Fisher, IN) was also determined at the same setting and converted to molecules of equivalent soluble fluorochrome (MESF). Antibody binding sites were then determined on a scatter plot based on the MESF values.

### 2.4. Mean Platelet Volume

PRP (10 mL) were mixed with 10 mL of isotonic isoflow solution (7.93 mg/mL NaCl and 0.4 mg/mL KCl, Beckman Coulter) containing 1% glutaraldehyde. A mean platelet volume (MPV) was determined on a Z2 Coulter particle counter (Beckman Coulter, Miami, FL, USA) according to the manufacturer’s instructions. 

### 2.5. Plasma Fibrinogen

Plasma fibrinogen levels were determined using an automatic blood coagulation system (Behring Coagulation System, New Castle, DE, USA) according to the manufacturer’s instructions. This assay measured the kinetics of converting soluble fibrinogen into insoluble fibrin by bovine thrombin, and fibrin generation was then measured by a fixed absorbance. 

### 2.6. L33P Gene Polymorphism of β3 

The β_3_ integrin gene carries a polymorphism at amino acid position 33 involving a leucine to proline substitution, and this polymorphism constitutes the human platelet alloantigens PlA1 and PlA2 [25,26]. The polymorphism was detected on all donors using an SNP method on a 7700 Sequence Detector (Applied Biosystems, Foster City, CA, USA). For this, leukocytes were collected from the interface between PRP and red blood cell layers and genomic DNA was extracted using a Qiagen DNA extraction kit (Qiagen, Valencia, CA, USA) according to the manufacturer’s instructions. A polymerase chain reaction (PCR) was set up containing 100 ng of genomic DNA, 2.5 pM of two fluorescence probes, and two primers spanning the polymorphic site. Two probes were allele-specific and tugged with two different fluorescence dyes (PLA1 probe, 5′ VIC-CCTGCCTC**T**GGGCTCACCTC-TAMRA and PLA2 probe, 5′ FAM-CCTGCCTC**C**GGGCTCACCT-TAMRA. The PCR reaction was set at 40 cycles with 15 sec at 95 °C for denaturing and 60 sec at 65 °C for annealing/extension.

### 2.7. Statistical Analysis

Regression analysis was used to determine correlations between two methods of clot retraction and correlations of clot retraction with levels of plasma fibrinogen, the number ofα_IIb_β_3_ molecules on platelet surfaces, and MPV. One-way ANOVA was used to compare the difference between multiple groups of variables, including clot retraction, among three different anticoagulants and to compare whole-blood and PRP clot retraction and clot retraction with different platelet counts, levels of plasma fibrinogen, and the number of α_IIb_β_3_ molecules on platelet surfaces. The difference between the PLA alleles and the percentage of clot retraction was tested by a Mann–Whitney U test before it failed normality test for data distribution. All values were expressed as mean ± SEM.

## 3. Results

All donors were healthy individuals defined as having no history of bleeding disorders or cardiovascular disease and free of drugs that affect platelet functions such as aspirin, ticlopidine, clopidogrel, and antibiotics for at least a week. The study recruited 53 donors, including 27 males (51.5%) and 26 females (48.5%). Baseline information on the subjects included in this study is presented in Table 1.

### 3.1. Clot Retraction

Whole-blood and PRP clot retractions were performed 3–6 times on each of the 14 individuals and the intra-donor coefficient of variation (CV) was determined to be 2.7–12.1% and 0.5–6%, respectively. The CV was significantly smaller for the PRP assay compared to testing whole blood samples (Student’s *t*-test, n = 14, *p* < 0.01). There was no gender difference in the percentage of clot retraction (78.0% vs. 77.3% for the whole blood assay; 88.8% vs. 88.5% for the PRP assay).

**Figure 1:**
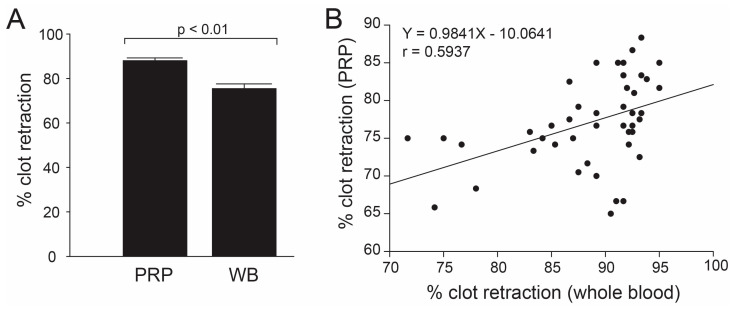
**Correlations between two methods for measuring clot retraction:** Whole blood assay (WB) was performed in the absence of anticoagulant and agonist, whereas the PRP assay (PRP) was induced by human α-thrombin using the citrated blood. The percentage of clot retraction was significantly less in the whole blood assay compared to the PRP assay ((**A**), Mann–Whitney U test, n = 53), and the results from the two methods were closely correlated ((**B**). Linear regression analysis, n = 53, *p* < 0.005).

**Figure 2:**
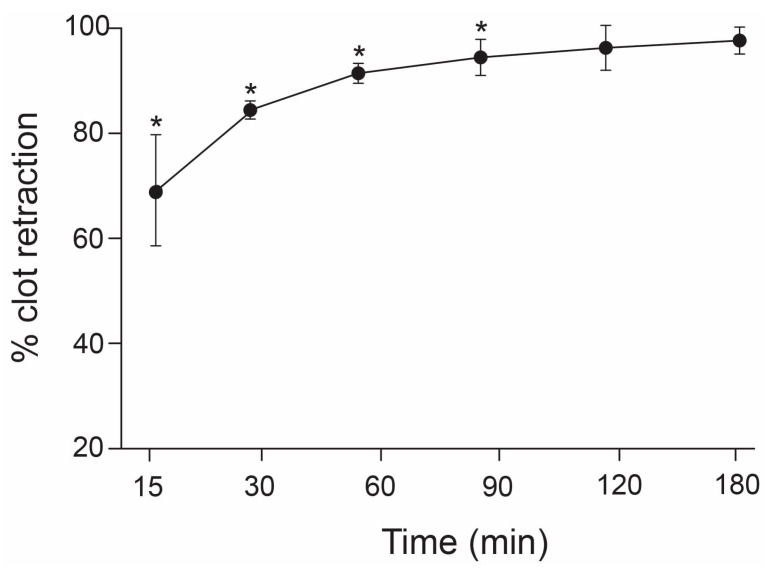
**Time course of the PRP clot retraction:** Aliquot of 0.6 mL of citrate PRP was incubated with 100 U of human α-thrombin for 15, 30, 60, 90, 120, and 180 min at 37 °C and clot retraction at each time point was measured. The percentage of clot retraction increased proportionally with the incubation time and reached the maximum at 90 min incubation (one-way ANOVA, * *p* < 0.05 compared to peak level at 180 min, n = 19).

To determine the effect of thrombin, clot retraction was performed using whole blood in the absence of thrombin and PRP in the presence of human α-thrombin. The percentage of clot retraction obtained from the whole blood assay was significantly less than that obtained from the PRP assay (Figure 1A), but results from the two assays were correlated (Figure 1A). The percentage of PRP clot retraction increased with increasing the incubation time from 30 to 90 min (74.4% vs. 90.6%, n = 19, *p* < 0.01) and remained constant thereafter (Figure 2).

### 3.2. Effects of Anticoagulants on Clot Retraction

**Figure 3:**
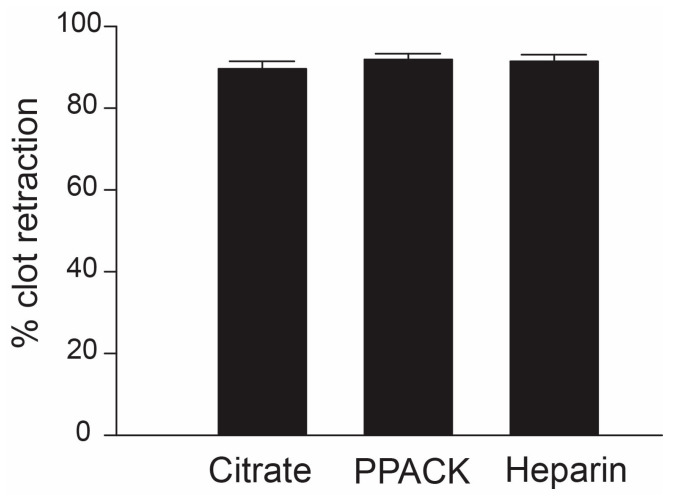
**Clot retraction with different anticoagulants**: Clot retraction was induced by incubating PRP with 100 U of human α-thrombin for 2 hrs at 37 °C using citrate, PPACK, or heparin as an anticoagulant. The percentage of clot retractions showed no significant differences between the three anticoagulants (one-way ANOVA, n = 14/group).

A comparison was also made among three anticoagulants: citrate, PPACK, and unfractionated heparin for their effects on clot retraction induced by thrombin in PRP. This comparison allowed us to determine the role of extracellular calcium in retracting clots because sodium citrate chelates and depletes extracellular calcium, while both PPACK and heparin maintain a physiological level of plasma calcium. In addition, heparin has been reported to reduce the size of fibrin fibers in a clot caused by heparin inhibition of thrombin activity [30]. However, we did not detect a difference in the rate of clot retraction in samples collected with the three anticoagulants (Figure 3).

### 3.3. Effects of Platelet Counts and Mean Platelet Volume on Clot Retraction

**Figure 4:**
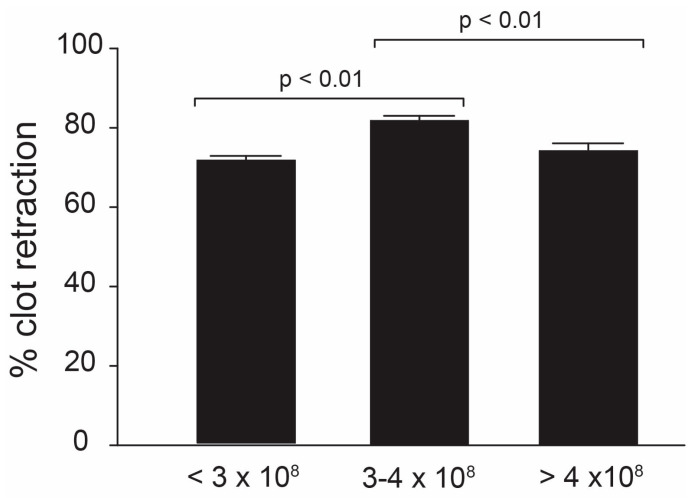
**Correlations between clot retraction and platelet counts**: Whole-blood clot retraction was measured and divided into three groups according to their platelet counts: counts of less than 3 × 10^8^/mL, counts between 3–4 × 10^8^/mL, and counts of more than 4 × 10^8^/mL. The percentage of clot retraction was significantly greater in samples whose platelet counts were between 3–4 × 10^8^/mL compared to those with either higher or lower platelet counts (one-way ANOVA, n = 14).

Because of its dependence on functional platelets, we determined the effect of platelet counts on clot retraction using whole-blood clot retraction, where platelet counts were not adjusted. We did not find an overall correlation between clot retraction and platelet counts (regression analysis, R^2^ = 0.003), but the percentage of clot retraction was greater in whole blood, with platelet counts of 300,000–400,000/μL compared to that with platelet counts either below or beyond this range (Figure 4, ANOVA, n = 14/group, *p* < 0.01).

Since large platelets are shown to be hemostatically more active and mean platelet volume is increased during acute myocardial infarction and unstable angina [31,32], we also correlated MPV with clot retraction. MPV ranged from 108.4 to 416.0 μm^3^/mL with a mean value of 230.8 ± 11.5 μm^3^/mL. There was no significant difference between males and females (219.6 ± 22.7 vs. 205.6 ± 16.8 μm^3^/mL, n = 53, *p* = 0.628). Clot retraction in whole blood was not associated with MPV (R^2^ = 0.0572, *p* = 0.203). 

### 3.4. Effects of Fibrinogen on Clot Retraction 

**Figure 5:**
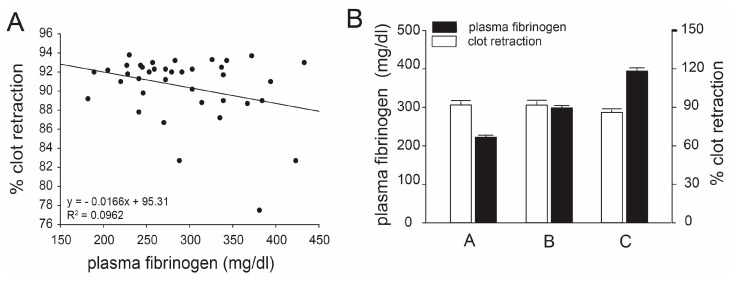
**Correlations between clot retraction and plasma levels of fibrinogen:** Plasma fibrinogen levels were measured for each donor by an automatic blood analyzer; no overall correlation was found with clot retraction (**A**, linear regression analysis, n = 33). Samples were further divided into three groups based on their fibrinogen levels: group A had fibrinogen levels less than 250 μg/mL; group B had fibrinogen levels between 250 and 350 μg/mL; group C had fibrinogen levels greater than 350 μg/mL. There was no significant difference in clot retraction among these three groups (**B**, one-way ANOVA, n = 33, *p* > 0.05).

Plasma levels of fibrinogen were from 182 to 433 mg/dl, determined using a kinetic assay, and were not associated with the percentage of clot retraction in whole blood (r^2^ = 0.0783, *p* = 0.571, Figure 5A), even after the subjects were grouped based on quartiles of plasma fibrinogen levels (Figure 5B).

### 3.5. Effect of Platelet α_IIb_β_3_ on Clot Retraction

**Figure 6:**
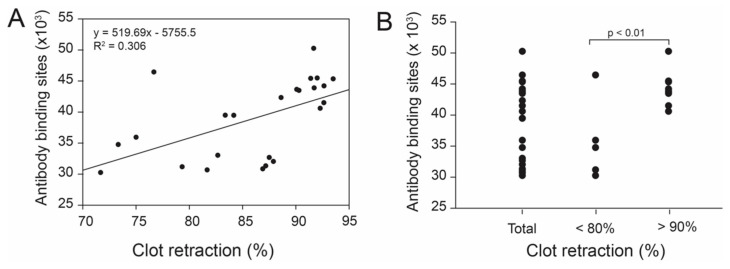
**Correlations between clot retraction and surface density of the platelet α_IIb_β_3_**: The numbers of α_IIb_β_3_ on platelet surfaces were determined by a flow cytometric method using the monoclonal antibody P2, which recognizes the α_IIb_β_3_ complex, but not individual subunits. There was no overall correlation between clot retraction and surface density of the platelet α_IIb_β_3_ (**A**, linear regression analysis, n = 24). The surface density of the α_IIb_β_3_ was, however, significantly greater in samples with clot retraction greater than 90% than those with clot retraction of less than 80% (**B**, Student’s *t*-test, n = 7–12).

The number of α_IIb_β_3_ receptor on platelet surfaces was measured using the monoclonal α_IIb_β_3_ antibody P2 and this number was not associated with clot retraction for the entire cohort of samples (regression analysis, r^2^ = 0.324, Figure 6A). However, the binding of the P2 antibody was significantly lower in individuals with less than 80% clot retraction than those with greater than 90% clot retraction (Figure 6B). 

### 3.6. PLA Genotypes and Clot Retraction

It has been reported that individuals carrying the PLA2 genotype have a higher risk of developing arterial thrombosis [33,34]. We genotyped 100 healthy subjects and determined the frequencies for the PLA1 homozygote, PLA1/PLA2 heterozygote, and PLA2 homozygote, which were 68, 28, and 4%, respectively, with 82% PLA1 allele carriers and 18% PLA2 carriers (Table 2), consistent with a previous report [35]. We did not detect a significant difference in clot retraction and P2 binding between donors who carried the PLA1 allele and those who carried the PLA2 allele (Table 2).

## 4. Discussion

Clot retraction is a process that requires functional platelets. This assay can therefore be used to detect platelet defects. However, it is unknown whether this assay can also be used to assess platelet hyper-reactivity, which is associated with arterial thrombosis. In an attempt to evaluate correlations between clot retraction and other indicators of platelet function, we performed clot retraction and platelet function assays on 53 healthy donors. The intra-donor CV for clot retraction was low, suggesting that the assay is highly reproducible. The intra-donor CV was lower in the PRP assay (0.5–6%) compared to the whole blood assay (2.7 vs. 12.1%). The former was induced by human α-thrombin in the presence of citrate, PPACK, or heparin, whereas the latter occurred spontaneously in the absence of an anticoagulant agent or platelet agonist. The serine protease thrombin cleaves fibrinogen into fibrin, which is then crosslinked into insoluble fibrin fibrils that stabilize a platelet clot and facilitate clot retraction to increase fibrin density [35]. Clot retraction is one of the limited numbers of tests to detect platelet contractile forces, which converts the dynamic mechanical microenvironments of circulation to biological responses due to bleeding [36]. By comparing results from the two standard clinical methods of clot retraction, we could therefore determine how exogenous thrombin affects the course of clot retraction. Thrombin is the end product of the coagulation cascade that leads to the cleavage of fibrinogen to fibrin and an agonist for platelet activation—both processes are critical for clot retraction. We found that the levels of clot retraction were greater in PRP, but results from two assays were closely correlated, suggesting that thrombin accelerated, but did not alter the course of clot retraction. Furthermore, the whole blood assay may have had a greater intra-donor CV but was likely to be sensitive to changes caused by pathological conditions.

Although calcium is required for the thrombin action in blood coagulation as well as in platelet activation, clot retraction was normal in PRP using either EDTA or sodium citrate as an anticoagulant [37]. This is consistent with our finding that the percentage of clot retraction was similar in PRP containing sodium citrate, PPACK, or unfractionated heparin (Figure 3). 

Because of the importance of the platelet α_IIb_β_3_ integrin in clot retraction and platelet aggregation, we next correlated clot retraction with functions of platelet α_Iib_β_3_, including the plasma levels of fibrinogen, surface density of the platelet α_Iib_β_3_ integrin, and PLA genotype. We found no overall correlation between clot retraction and plasma fibrinogen, the α_Iib_β_3_ density on platelet surfaces, or PLA genotypes. These results, however, do not exclude the effects of extraordinarily high or low levels of fibrinogen on clot retraction. Despite the lack of an overall correlation, the density of the α_Iib_β_3_ integrin was significantly greater in individuals whose clot retraction was greater than 90% compared to those with less than 80% of clot retraction, suggesting that a high surface density of the platelet α_Iib_β_3_ integrin may result in the formation of tighter clots. Such observations further highlight the clinical relevance of α_Iib_β_3_, where less than 80% of clot retraction is often indicative of deficient platelet contractile forces, while over 90% is normal. Interestingly, in contrast to α_Iib_β_3_ density, clot retraction was not correlated with platelet counts and mean platelet volume. The former showed a bell curve distribution with a maximal retraction occurring in samples containing 3–4 × 10^5^ platelets/μL (Figure 4). 

Recent evidence has also suggested the involvement of red blood cells (RBCs) in clot retraction. Primarily through their mechanical properties and a steric hindrance effect, RBCs can serve to resist clot retraction while transiently influencing platelet contractile forces [1]. Although we were unable to stratify for hematocrit in our studies, significant effects due to RBC are often observed in patients with very low (e.g., hemorrhagic shock) or very high (e.g., polycythemia) hematocrits. 

In summary, while clot retraction is less used in diagnosis, it has often been used to evaluate the effects of drugs on platelet contractile forces, which are critical for stabilizing a clot [6,38,39]. We measured clot retraction in 53 healthy donors and found that the assay was very reproducible. Clot retraction is affected by platelet counts, but not by mean platelet volume. It did not correlate with the plasma levels of fibrinogen but was affected by the surface density of α_IIb_β_3_ integrin. Finally, there was no difference in clot retraction between different PLA genotypes. These results indicate that clot retraction measures a unique aspect of platelet functions that could not be detected by the platelet functional assays that we performed.

## Figures and Tables

**Table 1:** Baseline information on subjects included in the study.

	White (31)	Black (11)	Asian (9)	Hispanic (2)
Female sex (%)	55	40	30	0
Age	46.2 ± 5.7	39.0 ± 2.6	29.0 ± 5.1	42
Platelet counts (10^3^/µL)	267 ± 132	311 ± 99	198 ± 89	298
RBC counts (10^4^/µL)	526 ± 219	499 ± 169	418 ± 176	498
Leukocyte counts (10^3^/µL)	5.6 ± 1.9	6.8 ± 3.9	6.3 ± 1.6	6.2

**Table 2:** Correlations between PLA genotypes and clot retraction.

	PLA1	PLA2
Allele frequency	41 (82%)	9 (18%)
Clot retraction (%)	88.18 ± 4.59	87.52 ± 6.13
P2 binding site	37,926 ± 5914	37,668 ± 6254

## Data Availability

The data presented in this study are available on request from the corresponding author.
